# Transition of care in pediatric surgery

**DOI:** 10.31744/einstein_journal/2021AO6314

**Published:** 2021-12-20

**Authors:** Amanda Botelho, Pedro Norton Gonçalves Dias, Andre Hiroki Suyama Tsuji, Mila Torii Corrêa Leite

**Affiliations:** 1 Escola Paulista Medicina Universidade Federal de São Paulo São Paulo SP Brazil Escola Paulista Medicina, Universidade Federal de São Paulo, São Paulo, SP, Brazil.

**Keywords:** Transitional care, Adolescent, Child, General surgery, Transition to adult care

## Abstract

**Objective:**

To identify among pediatric surgeons across Brazil how the transition of pediatric patients to adult clinics is carried out.

**Methods:**

A questionnaire was emailed to pediatric surgeons registered with the *Associação Brasileira de Cirurgia Pediátrica* in 2018. The data assessed included training time, maximum age of care, subspecialty of practice, outpatient follow-up of adult patients, reason for continuing care of adult patients, referral to adult specialties, concern with transition of care, and what has been done to improve it.

**Results:**

Most pediatric surgeons had more than 20 years of experience, and approximately 61% worked simultaneously at a public hospital, private hospital and private office. The maximum age of care at public, private hospitals and private offices proved to be quite varied. The follow-up of patients aged over 18 years at public hospitals, private hospitals and private clinics wase 32%, 23.58% and 20.75%, respectively. The main reason for patients aged over 18 years continued to be accompanied by pediatric surgeons was lack of knowledge about the disease by adult specialties. Most patients were referred to the adult specialty of the hospital, and roughly 37% of pediatric surgeons responded that they were in contact with the adult specialty. Most believed in autonomy of care of their patients and were concerned with transition of care.

**Conclusion:**

Transition of care is a relevant issue that needs to be studied and debated to ensure an appropriate long-term follow-up.

## INTRODUCTION

Pediatric surgery is a recent surgical specialty that treats patients within the age group of zero to 18 years. However, the complexity of the conditions, especially congenital diseases, demands longer follow-up. There is currently growing concern by several pediatric specialties about the best way to carry out the transition of these adolescent patients to adult settings. The number of publications on this subject has increased in recent years; however it is still little addressed, especially in surgical specialties, nationally or internationally.

The transition of care consists in the process of preparing referral of adolescents and young adults from pediatric care to adult settings. It includes initial planning, the transference itself, and the support provided throughout adulthood. The transition intends to prevent losing these patients to follow-up, what is frequently reported during this period, as well as to ensure autonomy of care. Unfortunately, the process is usually not well coordinated.^(1)^

## OBJECTIVE

To identify among pediatric surgeons across Brazil how the transition of pediatric patients to adult clinics is carried out.

## METHODS

The project was approved by the Research Ethics Committee of the *Universidade Federal de São Paulo* (UNIFESP), number 2.362.296, CAAE: 79343517.1.0000.5505. The survey was directed to pediatric surgeons throughout Brazil, of both sexes, who worked at public or private hospitals or/and private offices. A questionnaire prepared by the authors on a virtual platform was kindly emailed by the *Associação Brasileira de Cirurgia Pediátrica* [Brazilian Association of Pediatric Surgery] (CIPE) to registered pediatric surgeons from all over the country, in 2018 (Appendix 1). Those who did not complete the questionnaire in full were excluded from the analysis. A standardized Informed Consent Form (ICF) standardized by UNIFESP was available for reading at the beginning of the questionnaire, and its completion was considered as acceptance.

Data evaluated were the time since completion of training in the specialty (more than 20 years, 10 to 20 years and less than 10 years), state where they worked as pediatric surgeon, type of setting (public hospital, private hospital, private clinic), maximum patient age at the public and/or private hospital and private office, subspecialty, presence or absence of outpatient follow-up of patients older than 18 years, reason for continuing the follow-up of adult patients, how patients were referred to adult specialties, concern about the transition of care, and what had been done to improve transition of care.

For statistical analysis, variance analysis (Anova - *análise de variância*) was used to compare the groups in the Ghaph Pad Prism 8 software, with significance value set at p<0.05.

## RESULTS

About 800 emails were sent, and 106 questionnaires were filled in. The states that contributed with more questionnaires answered were São Paulo (28.30%), Paraná (11.32%) and Rio Grande do Sul (10.37%). About 65% had more than 20 years of experience in pediatric surgery, 22.64% had 10 to 20 years of experience and 13% less than 10 years (p<0.001).

**Figure 1:**
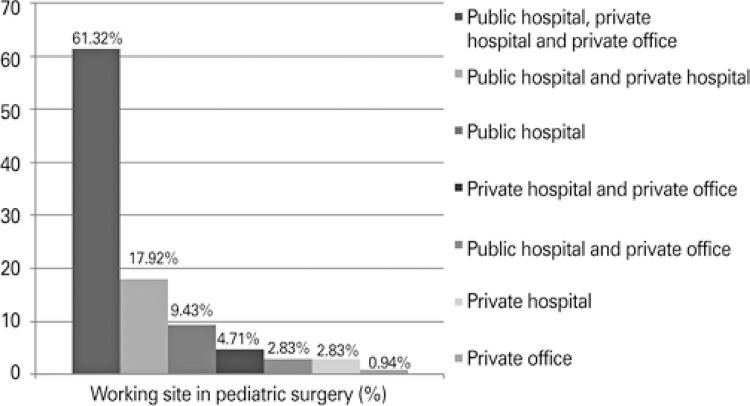
Working site in pediatric surgery

About 61.32% of physicians answered they worked simultaneously at public hospital, private hospital, and private office, 24.02% worked in two services, and a smaller percentage (13.20%) in only one service (p<0.001) (Figure 1).

The maximum age of care at public hospitals ranged from 12 years to 12 years and 11 months (27.35%); at private hospitals, from13 years and 11 months to 14 years and 11 months (24.52%); and at private offices from 17 years and 11 months to 18 years (19.8%). The comparison of these data was statistically significant (p<0.001) (Table 1).

**Table 1:** Maximum age in each service

Age	Public hospital	Private hospital	Private office
11 years and 11 months	4.71	2.83	1.88
12 years	10.37	9.43	6.60
12 years and 11 months	16.98	8.49	2.83
13 years	3.77	1.88	2.83
13 years and 11 months	3.77	9.43	5.66
14 years	4.71	9.43	6.60
14 years and 11 months	13.20	5.66	10.37
15 years	7.54	4.71	4.71
15 years and 11 months	4.71	2.83	1.88
16 years	1.88	5.66	4.71
16 years and 11 months	0.94	0.94	
17 years			0.94
17 years and 11 months	11.32	13.20	13.20
18 years	3.77	5.66	6.60
18 years and 11 months	0.94		
20 years	0.94		0.94
21 years	1.88	0.94	0.94
“No information”	6.59	10.37	17.92
“No age limit”	1.88	8.49	11.32

Results expressed as %.

Among the surgeons interviewed, 35.84% had more than one area of interest, and about 33% reported having no area of interest (p<0.001). The areas of interest mentioned were pediatric urology (67.64%), coloproctology (45.58%), thoracic surgery (32.35%), oncology (35.29%), and transplantation (7.35%).

**Figure 2:**
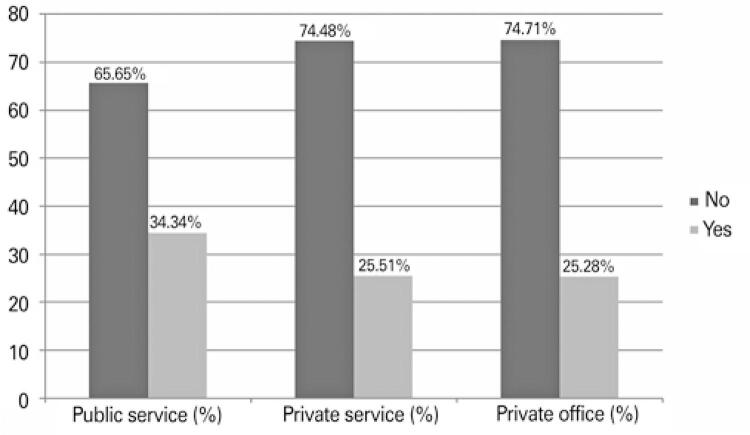
Care of patients aged over 18 years in each service

The outpatient follow-up of patients aged over 18 years at public hospitals, private hospitals and private office was, respectively, 34.34%, 25.51% and 25.28% (p<0.001) (Figure 2). When the responses of surgeons with and without subspecialty were analyzed, the percentage of patients aged over 18 years, cared for at public hospitals, private hospitals and private offices was, respectively, 38.09%, 27.77%, 26.98%, 22.85%, 31.57% and 13.79% (p=0.045).

The reasons why patients older than 18 years continued to be followed by pediatric surgeons in the services where they worked were, “does not apply, since I refer all patients older than 18 years” in 48.11% of the answers; “patient does not want to be referred to adult specialty” in 35.84% of the answers; “long-lasting physician-patient relationship, prevents referring to adult specialty” in 28.30% of the answers, and “lack of knowledge of this condition by adult specialties” in 22.64% of the answers (p<0.001) (Table 2).

**Table 2:** Reason to follow patients aged over 18 years

Reason	Responses
Does not apply since I refer all patients older than 18 years	48.11
Patient does not want to be referred to adult specialty	35.84
Long-standing physician-patient relationship, prevents referring to adult specialty	28.30
Adult specialties lack knowledge on this condition	22.64
Others	11.32

Results expressed as %.

Transition of patients to adult specialties was a current concern for 67.92% of the surgeons. A smaller percentage (29.24%), however, was not concerned about this issue (p<0.001). The concern about the transition among surgeons with and without area of interest was, respectively, 70.58% and 63.15% (p<0.001). About 85% of them answered the transition of patients to adult specialties could be improved (p<0.001).

Regarding the evaluation of the level of information and autonomy of their patients, 73.58% answered the patients were apt for self-care in adulthood. A total of 20.75% did not know how to answer this question, and only 5.66% reported that patients were not apt for self-care in adulthood (p<0.001).

Regarding the follow-up of patients in adulthood, 58.50% of physicians said they did not follow them in adulthood, 37.73% reported following through contact with specialists who provided care in adulthood, and 3.77% did not discharge patients (p<0.001).

The referral to the adult specialty occurred in several ways. About 64.15% reported that the patients were referred to the adult specialty of the hospital where they worked; 39.62% referred the patients to the health insurance adult specialty; 51.88% answered the patients were referred to a private adult specialty trusted by the surgeon; 17.92% reported the patients were referred to an adult specialty of another hospital, and 4.71% said they never referred the patients (p<0.001) (Table 3).

**Table 3:** Forms of referral to adult specialty

Forms of referral	Responses
Patient is referred to the adult specialty of the hospital where I work	64.15
Patient is referred to the private adult specialty I trust	51.88
Patient is referred to the health insurance adult specialty	39.62
Patient is referred to another hospital adult specialty	17.92
Does not apply because I never refer	4.71

Results expressed as %.

Regarding the measures pediatric surgeons took to carry out the transition of their patients, whether at public or private settings, 59% of responses indicated that nothing had been done, while 40% said that measures for the transition had been conducted, and a small percentage answered the question did not apply, since they followed the patients in adulthood (p<0.001). The adoption of measures related to the transition was answered as present by 28.94% of surgeons with no area of interest, and 45.58% of those with an area of interest (p<0.001). The measures taken for the transition are reported in the table 4.

**Table 4:** Pediatric surgeons’ responses on how they did patient transition

How do you carry out patient transition?
I try to follow along with the adult’s surgeon
Direct contact with the adult’s specialist
Orientations to patients and family members
I offer my opinion on management
Transition consultation to provide the patient all information and written documents on their problem and plan for the future
Make themselves available for the patient and adult specialist shall they have any doubt
Inform that the record is available at the hospital
Contact with the service and carry out procedures for referral
Refer for adult specialists with experience in congenital malformations
Send the physician a report with the patient treatment history
Project “*Travessia*” from the City Health Department and *Hospital Infantil Darcy Vargas,* in São Paulo
Detailed report given to legal guardians
Create transition groups

Regarding what could be accomplished to improve the transition of these patients to adult specialties, the main proposals were: training of adult specialists on these conditions, both at the private and public settings (65%), improving communication between physicians at private (31.13%) and public (45.28%) settings; improving patient’s education on their condition (40.56%), and outpatient departments with pediatric and adult specialists at public and private hospitals (38.67%) (p<0.001).

## DISCUSSION

Pediatric surgery is a new specialty. It emerged in the United States in the 1940´s and in Brazil in the 1960´s. Several congenital malformations have long survival and require care in adulthood.

In this research, we noticed great variation in the maximum age of care at public hospitals, private hospitals, and private offices. In public hospitals, care up to 12 years and 11 months prevails, while at the private offices, patients are treated with no restrictions until adulthood. The age group served at the organizations with medical residency may impact on the training of these specialists related to the clinical and surgical management of adolescents. Outpatient follow-up of patients aged over 18 years was found in both public and private services. In the Pediatric Urology Department of the *Escola Paulista de Medicina da* UNIFESP, in a recent unpublished survey, approximately 13% of adult patients continued to be followed up in the service. At The University of Virginia, in the United States, 20% of outpatient urological patients are aged over 18 years.^(2)^ The presence of adults seen in pediatric specialty outpatient clinics is frequent both at national and international levels. Several congenital and acquired malformations require clinical follow-up during childhood, adolescence and adulthood. In this survey, there was a higher prevalence of follow-up of patients aged over 18 years among surgeons with an area of interest, such as urology, coloproctology, thoracic surgery, oncology, and transplantation, both at public and private services. In these more specific areas of interest, there are patients whose clinical conditions require long-term follow-up. These surgeons reported being more concerned with the transition of their patients than those without an area of interest.

The main reason why patients aged over 18 years continued to be followed by pediatric surgeons in the services where they worked was the discomfort of the patient in not wanting to be referred to an adult specialty, followed by the long-established physician-patient relationship. In the literature, the unpreparedness of adult specialists to monitor congenital diseases or those acquired in childhood, associated with the unwillingness of pediatric health professionals to transfer their patients, and of patients to abandon the familiar environment established by the long bonding, are pointed out as factors that hinder this transition of care.^(3-5)^ Other gaps in supporting the transition are limited team training, lack of an identified team responsible for the transition, financial barriers, and anxiety on the part of pediatricians, adolescents and their parents about planning for their future health care.^(2,6)^

The responses showed most patients are referred to the adult specialty of the same hospital (public or private), and about 37% of pediatric surgeons answered they maintained contact with the adult specialty. The majority (about 73%) of surgeons answered they believed their patients were autonomous for self-care. The autonomy of care is one of the aspects analyzed in the questionnaires to evaluate preparedness for the transition.^(7,8)^ The family and professionals should encourage patients to gradually engage in their treatment until they acquire independence in care. They should be encouraged to know the name of the main drugs used and the reason why they are being administered, in addition to understanding and becoming more and more autonomous in the necessary procedures, such as intermittent bladder catheterizations and colonic cleansing.

The transition is often treated as an event, not as a gradual process of empowerment. Although most pediatric surgeons in this study have shown concern about the subject, the description of how they carry out the care transition showed its concept seems unclear. Studies have shown that some health professionals reported having transition agreements, but when examined more closely, it appears that they are simply transferring to adult services without training the patient or their caregiver.

There is evidence that morbidity and mortality increase in the change from pediatric to adult services, as well as poor compliance to treatment, increased readmission, clinical decompensation, and need for surgical reinterventions. Studies have shown an effective transition between pediatric and adult care improves long-term outcomes.^(7)^

Faced with this troubling scenario, the American Academy of Pediatrics (AAP), the American Academy of Family Physicians (AAFP) and the American College of Surgeons (ACS) issued a consensus statement, in 2002, which brought to surface the importance of supporting and facilitating transition care-services.^(9)^ An update was issued by the AAP in 2011, which provided careful guidance regarding the early preparation of children with chronic conditions for possible transition.^(2)^

In the United States, Canada and Europe, many hospitals have care transition programs.^(1)^ In England, there is a transition program called Ready Steady Go, implemented in all National Health Service (NHS) hospitals, aimed at patients aged over 11 years, and used for all pediatric subspecialties.^(7)^ In the visits, questionnaires with structured questions establishing the needs for adequate transfer to adult services are applied. Patients are encouraged to be seen without the presence of their caregivers at 13 and 14 years of age. At the age of 16 years, new questionnaires are applied to check autonomy of care is already guaranteed, and the first consultation with a team of the adult specialty is held. The transfer occurs only when the medical team, parents and the patient agree that it is the right time. An extensive report is then prepared by the pediatric team for transition of care. In the adult service, new questionnaires are applied to assess knowledge of self-care. In Brazil, we have few centers studying transition of care.

The limitation of this study was the small number of responses. Nevertheless, this research may reflect, even if partially, the scenario of this specialty. Research related to this topic is important and necessary to disseminate and expand the protocols of care and evaluation to other services.

## CONCLUSION

Transition of care for adult specialty still needs to be organized and standardized in most pediatric surgery services in Brazil.

It is a relevant subject that needs to be studied and debated, to ensure appropriate follow-up in the long run for patients seen in this pediatric specialty.

## Appendix 1

Questionnaire


