# Supplementary material for: Transition of care in pediatric surgery
Source: Einstein (Sao Paulo). 2021 Dec 20;19:eAO6314. doi: 10.31744/einstein_journal/2021AO6314 (PMC8693885; doi:10.31744/einstein_journal/2021AO6314)
Supplement: Supplementary file 1 [file 2317-6385-eins-19-eAO6314-suppl01.pdf]

**Appendix 1.** Questionnaire

1. In which state do you work as a pediatric surgeon?
2. For how long have you been working in pediatric surgery?
( ) More than 20 years
( ) 10-20 years
( ) Less than 10 years
3. Where do you practice pediatric surgery? (More than one answer possible)
( ) Private hospital
( ) Public hospital
( ) Private office
4. What is the maximum age of patients you see at the public hospital? ( )
( ) Does not apply
5. What is the maximum age of patients you see at the private hospital? ( )
( ) Does not apply
6. Until what age do you see patients in your private office? ( )
( ) Does not apply
7. Do you work in any pediatric surgery subspecialty?
( ) No
( ) Pediatric urology
( ) Coloproctology
( ) Transplant (kidney, liver, intestine)
( ) Oncology
( ) Thoracic surgery
8. Are patients aged over 18 years followed up in the same public service where you work?
( ) Yes
( ) No
( ) Does not apply
9. Are patients aged over 18 years followed up in the same private service where you work?
( ) Yes
( ) No
( ) Does not apply
10. Are patients aged over 18 years followed up in the same private office where you work?
( ) Yes
( ) No
( ) Does not apply
11. What is the reason for them to be followed up in the places where you work? (More than one answer possible)
( ) Lack of knowledge of this condition by the adult specialties
( ) Patient does not want referral to adult specialty
( ) Your long-lasting physician-patient relationship prevents referring the patient to the adult specialty
( ) Other __________
( ) Does not apply, since I refer all patients aged over 18 years
12. How do you make the referral to the adult specialty? (More than one answer possible)
( ) Does not apply since I never refer
( ) Patient is referred to the adult specialty of the hospital where I work
( ) Patient is referred to the adult specialty of another hospital
( ) Patient is referred to the health insurance adult specialty
( ) Patient is referred to the private adult specialty I trust
13. Do you follow the care provided to your patients as adults?
( ) Yes, I do not discharge the patients
( ) Yes, I keep contact with the adult specialties that follow them up
( ) No

continue...

...Continuation

**Appendix 1.** Questionnaire

14. Do you believe your patients are sufficiently informed and autonomous to care for themselves in adulthood?

( ) Yes

( ) No

15. Is your patient's transition to the adult specialty a current concern?

( ) Yes

( ) No

( ) Does not apply because I do not discharge these patients

16. Do you believe patients' transition to the specialties could improve?

( ) Yes

( ) No

17. What could be done to improve these patients' transition to adult specialties? (More than one answer possible)

( ) Does not apply since I will provide care for these patients in adulthood

( ) Improve communication between private practicing physicians

( ) Adult specialists trained to care for these conditions at private and public settings

( ) Outpatients clinics with pediatric and adult specialists at public hospitals

( ) Improve patient education on the condition

18. Have you been doing anything in your private or public practice to carry out these patients' transition?

( ) No

( ) Does not apply since I will provide care for these patients in adulthood

( ) Yes. What? ______
