# Supplementary material for: Transition of care in pediatric surgery
Source: Einstein (Sao Paulo). 2021 Dec 20;19:eAO6314. doi: 10.31744/einstein_journal/2021AO6314 (PMC8693885; doi:10.31744/einstein_journal/2021AO6314)
Supplement: Supplementary file 2 [file 2317-6385-eins-19-eAO6314-suppl01-pt.pdf]

Apêndice 1. Questionário

1. Qual estado que atua como cirurgião pediátrico?
2. Quanto tempo de atuação em cirurgia pediátrica?
( ) Mais de 20 anos
( ) 10-20 anos
( ) Menos de 10 anos
3. Onde atende cirurgia pediátrica? (Opção poderá ser múltipla)
( ) Hospital privado
( ) Hospital público
( ) Clínica privada
4. Qual a idade máxima que atende no hospital público que trabalha? ( )
( ) Não se aplica
5. Qual a idade máxima que atende no hospital privado que trabalha? ( )
( ) Não se aplica
6. Qual a idade máxima que atende na clínica privada que trabalha? ( )
( ) Não se aplica
7. Atua em alguma subespecialidade da cirurgia pediátrica?
( ) Não
( ) Urologia pediátrica
( ) Coloproctologia
( ) Transplante (renal, hepático, intestinal)
( ) Oncologia
( ) Torácico
8. Os pacientes acima de 18 anos continuam sendo atendidos no serviço público em que trabalha?
( ) Sim
( ) Não
( ) Não se aplica
9. Os pacientes acima de 18 anos continuam sendo atendidos no serviço privado em que trabalha?
( ) Sim
( ) Não
( ) Não se aplica
10. Os pacientes acima de 18 anos continuam sendo atendidos na clínica privada em que trabalha?
( ) Sim
( ) Não
( ) Não se aplica
11. Qual o motivo de continuarem sendo atendido nos serviços em que trabalha? (Opções podem ser múltiplas)
( ) Falta de conhecimento desta patologia pelas especialidades adultas
( ) Paciente não quer ser encaminhado para a especialidade adulta
( ) Sua longa relação médico-paciente não permite que consiga encaminhar para especialidade adulta
( ) Outra __________
( ) Não se aplica, pois encaminho todos os pacientes após 18 anos de idade
12. Como realiza o encaminhamento para especialidade adulta? (Opções podem ser múltiplas)
( ) Não se aplica porque nunca encaminho
( ) Paciente é encaminhado para a especialidade adulta do próprio hospital que trabalho
( ) Paciente é encaminhado para a especialidade adulta de outro hospital
( ) Paciente é encaminhado para a especialidade adulta do convênio
( ) Paciente é encaminhado para a especialidade adulta privada da minha confiança
13. Acompanha o seguimento na vida adulta de seus pacientes?
( ) Sim, não dou alta para os pacientes
( ) Sim, mantenho contato com as especialidades adultas que os acompanham
( ) Não

continua...

...Continuação

Apêndice 1. Questionário

14. Acredita que seus pacientes estão suficientemente informados e autônomos para manter seus cuidados na vida adulta?
( ) Sim
( ) Não
15. A transição dos seus pacientes para a especialidade adulta é uma preocupação atual para você?
( ) Sim
( ) Não
( ) Não se aplica porque não dou alta para estes pacientes
16. Acredita que a transição dos pacientes para as especialidades poderia melhorar?
( ) Sim
( ) Não
17. O que poderia ser realizado para melhorar a transição destes pacientes para as especialidades adultas? (Opções podem ser múltiplas)
( ) Não se aplica porque cuidarei dos pacientes na vida adulta
( ) Melhorar a interlocução entre os médicos da rede privada
( ) Especialistas adultos capacitados para atender a essas patologias na rede privada ou pública
( ) Ambulatórios com especialistas pediátricos e adultos nos hospitais públicos
( ) Melhorar a informação aos pacientes sobre sua patologia
18. Tem realizado alguma medida em sua atuação pública ou privada para realizar a transição destes pacientes?
( ) Não
( ) Não se aplica, acompanho todos meus pacientes na vida adulta
( ) Sim. Qual? _____
