# Supplementary material for: Transition of care in pediatric surgery
Source: Einstein (Sao Paulo). 2021 Dec 20;19:eAO6314. doi: 10.31744/einstein_journal/2021AO6314 (PMC8693885; doi:10.31744/einstein_journal/2021AO6314)
Supplement: Translation

# Translation: Transição de cuidados na cirurgia pediátrica

**Authors:** Amanda Botelho, Pedro Norton Gonçalves Dias, Andre Hiroki Suyama Tsuji, Mila Torii Corrêa Leite

**Affiliations:** 1 Brasil Escola Paulista Medicina, Universidade Federal de São Paulo, São Paulo, SP, Brasil.

**Keywords:** Cuidado transicional, Adolescente, Criança, Cirurgia geral, Transição para assistência do adulto

## Abstract

**Objetivo:**

Identificar entre os cirurgiões pediátricos de todo o Brasil como é realizada a transição de cuidados dos pacientes pediátricos para as clínicas adultas.

**Métodos:**

Um questionário foi enviado por *e-mail* para cirurgiões pediátricos cadastrados na Associação Brasileira de Cirurgia Pediátrica no ano de 2018. Foram avaliados dados como tempo de formação, idade máxima de atendimento, subespecialidade de atuação, seguimento ambulatorial de pacientes adultos, motivo pelo qual continuava atendendo pacientes adultos, encaminhamento para especialidades adultas, preocupação com a transição de cuidados e o que tinha realizado para aprimorá-la.

**Resultados:**

A maioria dos cirurgiões pediátricos tinha mais de 20 anos de experiência, e cerca de 61% atuavam simultaneamente em hospital público, hospital privado e clínica privada. A idade máxima de atendimento nos hospitais públicos e privados e nas clínicas privadas mostrou-se bastante variada. O acompanhamento de pacientes acima de 18 anos nos hospitais públicos, nos hospitais privados e nas clínicas privadas foi, respectivamente, 32%, 23,58% e 20,75%. O principal motivo pelo qual o paciente acima de 18 anos continuava sendo acompanhado por cirurgiões pediátricos foi falta de conhecimento da patologia pelas especialidades adulta. A maioria dos pacientes era encaminhada para a especialidade adulta do próprio hospital, e cerca de 37% dos cirurgiões pediátricos responderam que mantinham contato com a especialidade adulta. A maioria acreditava na autonomia de cuidados de seus pacientes e estava preocupada com a transição de cuidados.

**Conclusão:**

A transição de cuidados é um assunto relevante e que precisa ser estudado e debatido para que se possa assegurar um adequado seguimento a longo prazo.

## INTRODUÇÃO

A cirurgia pediátrica é uma especialidade cirúrgica recente, que atende pacientes dentro da faixa etária de zero a 18 anos. No entanto, a complexidade das patologias, principalmente as congênitas, requer um acompanhamento mais prolongado. Atualmente, há uma preocupação crescente de diversas especialidades pediátricas sobre a melhor maneira de realizar a transição desses pacientes adolescentes para as clínicas adultas. O número de publicações sobre esse tema vem aumentando nos últimos anos, mas ainda se trata de assunto pouco abordado, principalmente nas especialidades cirúrgicas, seja nacional ou internacionalmente.

A transição de cuidados consiste no processo de preparação da transferência de adolescentes e jovens adultos das clínicas de atendimento pediátrico para as de adulto. Inclui o planejamento inicial, a própria transferência e o suporte fornecido em todo o atendimento ao adulto. A transição visa prevenir a perda de seguimento desses pacientes, frequentemente relatada nesse período, e garantir a autonomia de cuidados. Infelizmente, o processo geralmente é pouco coordenado.^(1)^

## OBJETIVO

Identificar entre os cirurgiões pediátricos de todo o Brasil como é realizada a transição de cuidados dos pacientes pediátricos para as clínicas adultas.

## MÉTODOS

O projeto foi aprovado pelo Comitê de Ética em Pesquisa (CEP) da Universidade Federal de São Paulo (UNIFESP), número 2.362.296, CAAE: 79343517.1.0000.5505. A pesquisa foi destinada a médicos cirurgiões pediátricos de todo o Brasil, de ambos os sexos, que atendessem em hospitais público ou privados ou/e clínicas privadas. Um questionário elaborado pelos próprios autores em uma plataforma virtual foi gentilmente enviado por *e-mail* pela Associação Brasileira de Cirurgia Pediátrica (CIPE) para cirurgiões pediátricos cadastrados de todo o país no ano de 2018 (Apêndice 1). Foram excluídos da análise aqueles que não preencheram o questionário de maneira integral. Um Termo de Consentimento Livre e Esclarecido (TCLE) padronizado pela UNIFESP estava disponível para leitura no início do questionário, e seu preenchimento foi considerado como aceito.

Foram avaliados dados como o tempo de formação na especialidade (mais de 20 anos, 10 a 20 anos e menos de 10 anos), Estado onde atuava como cirurgião pediátrico, que tipo de atendimento realizava (hospital público, hospital privado, clínica privada), qual a idade máxima que atendia no hospital público e/ou privado e clínica privada, qual a subespecialidade de atuação, presença ou ausência de seguimento ambulatorial de pacientes maiores de 18 anos, o motivo pelo qual continuava realizando o seguimento de pacientes adultos, a maneira como referenciava seus pacientes às especialidades adultas, preocupação com a transição de cuidados e o que tinha realizado para aprimorar a transição de cuidados.

Para a análise estatística, foi utilizada a análise de variância (Anova) para comparação entre os grupos no software Ghaph Pad Prism 8, sendo utilizado o valor de significância de p<0,05.

## RESULTADOS

Foram enviados cerca de 800 *e-mails*, e foram preenchidos 106 questionários. Dentre os questionários preenchidos, os estados que contribuíram com mais respostas foram São Paulo (28,30%), Paraná (11,32%) e Rio Grande do Sul (10,37%). Cerca de 65% apresentavam mais de 20 anos de experiência em cirurgia pediátrica, 22,64% tinham de 10 a 20 anos de experiência e 13% menos de 10 anos (p<0,001).

Cerca de 61,32% dos médicos responderam que atuavam simultaneamente em hospital público, hospital privado e clínica privada, 24,02% atuavam em dois serviços, e uma menor percentagem (13,20%) atuava em apenas um serviço (p<0,001) (Figura 1).

**Figura 1:** Local de atendimento de cirurgia pediátrica

Os intervalos de idade máxima mais atendidos no hospital público foram de 12 anos a 12 anos e 11 meses (27,35%); 13 anos e 11 meses a 14 anos e 11 meses no hospital privado (24,52%) e 17 anos e 11 meses a 18 anos (19,8%) na clínica privada. Houve significância estatística na comparação entre esses dados (p<0,001) (Tabela 1).

**Tabela 1:** Idade máxima em cada serviço

Idade	Hospital público	Hospital privado	Clínica privada
11 anos e 11 meses	4,71	2,83	1,88
12 anos	10,37	9,43	6,60
12 anos e 11 meses	16,98	8,49	2,83
13 anos	3,77	1,88	2,83
13 anos e 11 meses	3,77	9,43	5,66
14 anos	4,71	9,43	6,60
14 anos e 11 meses	13,20	5,66	10,37
15 anos	7,54	4,71	4,71
15 anos e 11 meses	4,71	2,83	1,88
16 anos	1,88	5,66	4,71
16 anos e 11 meses	0,94	0,94	
17 anos			0,94
17 anos e 11 meses	11,32	13,20	13,20
18 anos	3,77	5,66	6,60
18 anos e 11 meses	0,94		
20 anos	0,94		0,94
21 anos	1,88	0,94	0,94
“Sem dados”	6,59	10,37	17,92
“Não há limite de idade”	1,88	8,49	11,32

Resultados expressos como %.

Dentre os cirurgiões entrevistados, 35,84% tinham mais de uma área de interesse, e cerca de 33% referiram não ter nenhuma área de interesse (p<0,001). As áreas de interesses apontadas foram urologia pediátrica (67,64%), coloproctologia (45,58%), cirurgia torácica (32,35%), oncologia (35,29%) e cirurgia de transplante (7,35%).

O acompanhamento ambulatorial de pacientes acima de 18 anos nos hospitais públicos, nos hospitais privados e nas clínicas privadas foi, respectivamente, de 34,34%, 25,51% e 25,28% (p<0,001) (Figura 2). Quando avaliada as respostas de cirurgiões com e sem subespecialidade, o atendimento de pacientes acima de 18 anos nos hospitais públicos, nos hospitais privados e nas clínicas privadas foi, respectivamente, de 38,09%, 27,77%, 26,98%, 22,85%, 31,57% e 13,79% (p=0,045).

**Figura 2:** Atendimento de pacientes acima de 18 anos em cada serviço

Os motivos pelos quais os pacientes acima de 18 anos continuavam sendo acompanhados por cirurgiões pediátricos nos serviços onde trabalhavam foram “não se aplica, pois encaminho todos os pacientes após 18 anos de idade” em 48,11% das respostas; “paciente não quer ser encaminhado para a especialidade adulta” em 35,84% das respostas; “longa relação médico-paciente não permite que consiga encaminhar para especialidade adulta” em 28,30% das respostas e “falta de conhecimento desta patologia pelas especialidades adultas” em 22,64% das respostas (p<0,001) (Tabela 2).

**Tabela 2:** Motivo do acompanhamento de pacientes acima dos 18 anos

Motivos	Respostas
Não se aplica, pois encaminho todos os pacientes após 18 anos de idade	48,11
Paciente não quer ser encaminhado para a especialidade adulta	35,84
Sua longa relação médico-paciente não permite que consiga encaminhar para especialidade adulta	28,30
Falta de conhecimento desta patologia pelas especialidades	22,64
Outros	11,32

Resultados expressos como %.

Alegaram que a transição de seus pacientes para a especialidade adulta era uma preocupação atual 67,92% dos cirurgiões. Uma menor parte (29,24%), no entanto, não se preocupava com tal questão (p<0,001). A preocupação com a transição entre os cirurgiões com e sem área de interesse foi, respectivamente, 70,58% e 63,15% (p<0,001). Cerca de 85% deles responderam que a transição dos pacientes para a especialidade adulta poderia melhorar (p<0,001).

Em relação à avaliação quanto à informação e autonomia de seus pacientes, 73,58% responderam que seus pacientes tinham condições de manter os cuidados na vida adulta. Os 20,75% não souberam responder à essa pergunta e apenas 5,66% referiram que os pacientes não estavam aptos a manter os cuidados na vida adulta (p<0,001).

Em relação ao seguimento dos pacientes na vida adulta, 58,50% dos médicos disseram não acompanhá-los na vida adulta, 37,73% referiram acompanhá-los por meio de contato com os especialistas que os seguiam na vida adulta e 3,77% não davam alta para os pacientes (p<0,001).

O encaminhamento para a especialidade adulta se dava de diversas formas. Cerca de 64,15% referiram que o paciente era encaminhado para a especialidade adulta do próprio hospital onde trabalhava; 39,62% encaminhavam o paciente para a especialidade adulta do convênio; 51,88% responderam que o paciente era encaminhado para a especialidade adulta privada de confiança do cirurgião; 17,92% referiram que o paciente era encaminhado para especialidade adulta de outro hospital e 4,71% disseram que nunca encaminhavam o paciente (p<0,001) (Tabela 3).

**Tabela 3:** Forma de encaminhamento para especialidade adulta

Forma de encaminhamento	Respostas
Paciente é encaminhado para especialidade adulta do próprio hospital que trabalho	64,15
Paciente é encaminhado para especialidade adulta privada da minha confiança	51,88
Paciente é encaminhado para especialidade adulta do convênio	39,62
Paciente é encaminhado para especialidade adulta de outro hospital	17,92
Não se aplica porque nunca encaminho	4,71

Resultados expressos como %.

Em relação às medidas que os cirurgiões pediátricos tomavam para realizar a transição de seus pacientes, seja na rede pública ou privada, 59% das respostas apontavam que nada tinha sido feito, enquanto 40% disseram que medidas para a transição tinham sido realizadas, e pequena percentagem respondeu que a pergunta não se aplicava, porque acompanhava os pacientes na vida adulta (p<0,001). A adoção de medidas relacionadas à transição foi respondida como presente em 28,94% dos cirurgiões sem área de interesse e em 45,58% daqueles com área de interesse (p<0,001). As medidas realizadas para a transição estão reportadas na tabela 4.

**Tabela 4:** Respostas dos cirurgiões pediátricos sobre como realizam a transição dos pacientes

Como realizam a transição dos pacientes?
Procuro acompanhar em conjunto com o cirurgião adulto
Contato direto com o especialista de adulto
Orientações aos familiares e pacientes
Ofereço a minha opinião quanto à conduta
Consulta de transição para dar ao paciente todas as informações e documentos escritos sobre seu problema e plano para o futuro
Colocar-se à disposição do paciente e do especialista adulto para qualquer dúvida
Informar que o prontuário está disponível no hospital
Contato com serviço e realizar os procedimentos para o encaminhamento
Encaminhar para especialistas adultos experientes em malformações congênitas
Enviar relatório com histórico do tratamento do paciente direcionado ao médico
Projeto Travessia da Secretaria de Saúde e Hospital Infantil Darcy Vargas de São Paulo
Relatório detalhado nas mãos dos responsáveis legais
Criar os grupos de transição

No que diz respeito ao que poderia ser realizado para melhorar a transição destes pacientes para as especialidades adultas, foram as principais propostas: capacitação dos especialistas adultos para atender essas patologias na rede privada ou pública (65%), melhora da interlocução entre os médicos da rede privada (31,13%) e na rede pública (45,28%); melhoraria da informação aos pacientes sobre sua patologia (40,56%) e ambulatórios com especialistas pediátricos e adultos nos hospitais públicos ou privados (38,67%) (p<0,001).

## DISCUSSÃO

A cirurgia pediátrica é uma especialidade recente. Ela surgiu nos Estados Unidos a partir da década de 1940 e, no Brasil, na década de 1960. Diversas malformações congênitas apresentam longa sobrevida e necessitam de cuidados na vida adulta.

Nesta pesquisa, observou-se grande variação na idade máxima de atendimento nos hospitais públicos, nos hospitais privados e nas clínicas privadas. Nos hospitais públicos, os atendimentos até 12 anos e 11 meses são os mais prevalentes, enquanto na clínica privada os pacientes são atendidos sem restrições até a vida adulta. A faixa etária atendida nos serviços em que tenha residência médica pode impactar na formação destes especialistas quanto ao manejo clínico e cirúrgico do adolescente. O acompanhamento ambulatorial de pacientes com idade acima de 18 anos foi observado tanto nos serviços públicos como nos serviços privados. No setor de Urologia Pediátrica da Escola Paulista de Medicina da UNIFESP, em pesquisa recente não publicada, identificou-se que cerca de 13% de pacientes adultos continuavam sendo acompanhados no ambulatório. Na Universidade da Virgínia, nos Estados Unidos, 20% dos pacientes urológicos ambulatoriais também são maiores de 18 anos.^(2)^ A presença de adultos atendidos nos ambulatórios de especialidades pediátricas é frequente tanto nacional como internacionalmente. Diversas malformações congênitas e adquiridas necessitam de seguimento clínico durante a infância, a adolescência e a vida adulta. Nesta pesquisa, houve maior prevalência de acompanhamento de pacientes com idade acima de 18 anos entre os cirurgiões com área de interesse, como urologia, coloproctologia, cirurgias torácica, oncológica e transplante de órgão, tanto nos serviços públicos como nos privados. Nessas áreas de interesse mais específicas, há pacientes cuja condições clínicas exigem um seguimento a longo prazo. Esses cirurgiões responderam estar mais preocupados com a transição de seus pacientes do que aqueles sem área de interesse.

O principal motivo pelo qual o paciente acima de 18 anos continuava sendo acompanhado por cirurgiões pediátricos nos serviços onde eles trabalhavam foi o desconforto do paciente em não querer ser encaminhado a uma especialidade adulta, seguido da longa relação médico paciente estabelecida. Na literatura, o despreparo de especialistas adultos em acompanhar patologias congênitas ou adquiridas na infância, associado à relutância dos próprios profissionais de saúde pediátricos em transferir seus pacientes e a dos pacientes em abandonar o ambiente familiar estabelecido pelo longo vínculo, são apontados como fatores que prejudicam essa transição de cuidados.^(3-5)^Outras lacunas no apoio à transição são o treinamento limitado da equipe, a falta de equipe identificada responsável pela transição, as barreiras financeiras e a ansiedade por parte de pediatras, adolescentes e seus pais sobre o planejamento para seus futuros cuidados de saúde.^(2,6)^

As respostas revelaram que a maioria dos pacientes é encaminhada para a especialidade adulta do próprio hospital (público ou privado), e cerca de 37% dos cirurgiões pediátricos responderam que mantinham contato com a especialidade adulta. A maioria (cerca de 73%) dos cirurgiões respondeu que acreditava na autonomia de cuidados de seus pacientes. A autonomia de cuidados é um dos quesitos analisados nos questionários de avaliação do preparo para a transição.^(7,8)^A família e os profissionais devem estimular os pacientes a gradualmente se envolverem com seu tratamento, até adquirirem independência de cuidados. Eles devem ser estimulados a saber o nome dos principais medicamentos utilizados e o motivo pelo qual eles estão sendo administrados, além de compreender e realizar cada vez com mais autonomia os procedimentos necessários, como cateterismos vesicais intermitentes e lavagens intestinais.

Muitas vezes, a transição é tratada como um evento, e não como um processo gradual de empoderamento. Apesar de a maioria dos cirurgiões pediátricos neste estudo ter demonstrado preocupação com o tema, a descrição de como eles realizam a transição de cuidados revelou que seu conceito parece pouco claro. Os estudos têm demonstrado que alguns profissionais de saúde relatam ter acordos de transição, mas, quando examinados mais de perto, parece que eles estão simplesmente transferindo para serviços de adultos sem a capacitação do jovem ou de seu cuidador.

Há evidências de que há aumento da morbidade e da mortalidade na mudança de serviços pediátricos para adulto, má aderência ao tratamento, aumento de reinternações, descompensações clínicas e necessidades de reintervenções cirúrgicas. Estudos mostram que uma transição efetiva entre cuidados pediátricos e adultos melhora os resultados a longo prazo.^(7)^

Diante desse cenário preocupante, a *American Academy of Pediatrics* (AAP), a *American Academy of Family Physicians* (AAFP) e o *American College of Surgeons* (ACS) emitiram uma declaração de consenso, em 2002, que trouxe à tona a importância de apoiar e facilitar os serviços de atendimento de transição.^(9)^ Uma atualização foi emitida pela AAP em 2011, a qual oferece orientações cuidadosas com relação à preparação precoce de crianças com condições crônicas para eventual transição.^(2)^

Nos Estados Unidos, Canadá e Europa, muitos hospitais apresentam programas de transição de cuidados.^(1)^ Na Inglaterra, há um programa de transição, chamado *Ready Steady Go*, implementado em todos os hospitais do *National Health Service* (NHS) direcionado aos pacientes acima de 11 anos e usado para todas as subespecialidades pediátricas.^(7)^ Nas consultas, são aplicados questionários com questões estruturadas que estabelecem as necessidades para uma adequada transferência aos serviços adultos. Os pacientes são encorajados a serem atendidos sem a presença de seus cuidadores aos 13 e 14 anos. Aos 16 anos, novos questionários são aplicados, para a checagem de que a autonomia dos cuidados já está garantida, e é realizada a primeira consulta com equipe da especialidade adulta. A transferência ocorre somente quando a equipe médica, os pais e o próprio paciente concordam que seja o momento adequado. Um relatório extenso é, então, realizado pela equipe pediátrica, para a transição de cuidados. No serviço adulto, novos questionários são aplicados para a avaliação do conhecimento do autocuidado. No Brasil, temos poucos centros estudando a transição de cuidados.

A limitação deste trabalho foi o número reduzido de respostas. Mesmo assim, esta pesquisa pode retratar, ainda que parcialmente, o cenário desta especialidade. Pesquisas relacionadas a esse tema são importantes e necessárias para que os protocolos de atendimento e de avaliação sejam divulgados e expandidos para outros serviços.

## CONCLUSÃO

A transição de cuidados para a especialidade adulta ainda necessita de organização e padronização na maioria dos serviços de cirurgia pediátrica no Brasil.

Trata-se de um assunto relevante, que precisa ser estudado e debatido, para que se possa assegurar um adequado seguimento em longo prazo dos pacientes atendidos nessa especialidade pediátrica.

## Apêndice 1

Questionário
